# Keeping the Aging Brain Healthy through Exercise

**DOI:** 10.3390/brainsci12060717

**Published:** 2022-05-31

**Authors:** Notger G. Müller

**Affiliations:** 1 Research Group Degenerative and Chronic Diseases, Exercise, Faculty of Health Sciences, University of Potsdam, 14476 Potsdam, Germany; notger.mueller@uni-potsdam.de; 2 Research Group Neuroprotection, German Centre of Neurodegenerative Diseases, 39120 Magdeburg, Germany

The interaction of physical activity and brain function with respect to what we now call successful aging has been and remains extensively studied. In general, a wealth of studies indicates that short- and long-term physical activity can induce neuroplasticity even in the adult brain, can enhance cognitive performance positively and may reduce the risk of neurodegenerative diseases. However, the underlying neurobiological mechanisms of physical activity on the human central nervous systems are not yet fully understood. Additionally, what type of exercise might be optimal for keeping the brain fit in old age and whether imagined as opposed to real exercise has the potential to be effective as well is not yet clear. In this Special Issue of *Brain Sciences*, six high-quality articles assess the mentioned open questions:

In their paper “Increased Time Difference between Imagined and Physical Walking in Older Adults at a High Risk of Falling”, Nakano et al. demonstrate that older adults who are at a higher risk of falling show longer walking times during action execution but believe that they would be faster during imagined walking [1]. The authors conclude that the time difference between imagined and real walking could be useful for assessing the risk of falling in seniors.

In their paper “Timing-Dependent Protection of Swimming Exercise against d-Galactose-Induced Aging-Like Impairments in Spatial Learning/Memory in Rats”, Li et al. observed that swimming exercise training in rats whose aging process was artificially enhanced by drugs protected the rats’ hippocampal neurons against degenerative changes and maintained neuronal synaptic plasticity and memory function [2]. The authors also speculate about the molecular mechanisms of the named neuroprotective effects induced by swimming.

In their paper “Superior Effects of Modified Chen-Style Tai Chi versus 24-Style Tai Chi on Cognitive Function, Fitness, and Balance Performance in Adults over 55”, Zou et al. report that two forms of Tai Chi training enhanced global cognitive function, balance, and fitness [3]. The modified training program with additional exercises, however, showed more pronounced effects than the more traditional training.

In their paper “The Contribution of Functional Magnetic Resonance Imaging to the Understanding of the Effects of Acute Physical Exercise on Cognition”, Herold et al. review studies that used functional magnetic resonance imaging (fMRI) to investigate the effects of acute physical exercising on cerebral hemodynamics and cognition [4]. They identify limitations of these former studies and demand that future studies should use more rigorous study designs, apply more sophisticated filter methods in fMRI data analysis, describe the applied processing steps of fMRI data analysis in more detail, and provide a more precise exercise prescription.

In their paper “Mental Imagery and Acute Exercise on Episodic Memory Function” Johnson et al. aimed to evaluate whether acute exercise and mental imagery of acute exercise have similar effects on cognitive performance, specifically memory function. They found that both actual exercising and imagined exercising affect memory function to the same extent [5]. This is positive news for people whose ability to exercise in real life is limited.

In their paper “Working Memory, Cognitive Load and Cardiorespiratory Fitness: Testing the CRUNCH Model with Near-Infrared Spectroscopy”, Agbangla et al. found that among older adults, those with higher levels of cardiorespiratory fitness had increased bilateral prefrontal cortex activation and better cognitive performance, especially under the highest cognitive load, a finding which is in line with the neurocognitive CRUNCH model [6].

In sum, this Special Issue offers reports of exciting studies on the interaction of exercise and brain health. Nevertheless, further efforts will be necessary in the future to complete the emerging picture of this interaction.
